# Decoding LINC00052 role in breast cancer by bioinformatic and experimental analyses

**DOI:** 10.1080/15476286.2024.2355393

**Published:** 2024-06-04

**Authors:** Jose Manuel Sanchez-Lopez, Miguel Angel Juarez-Mancera, Benjamin Bustamante, Araceli Ruiz-Silvestre, Magali Espinosa, Gretel Mendoza-Almanza, Gisela Ceballos-Cancino, Jorge Melendez-Zajgla, Vilma Maldonado, Floria Lizarraga

**Affiliations:** a Laboratorio de Epigenetica, Instituto Nacional de Medicina Genomica (INMEGEN), Ciudad de México, Mexico; b Laboratorio de Genomica Funcional del Cancer, Instituto Nacional de Medicina Genomica (INMEGEN), Ciudad de México, Mexico

**Keywords:** LINC00052, breast cancer, lncRNA, DNA damage, Estrogen receptor

## Abstract

LncRNA is a group of transcripts with a length exceeding 200 nucleotides that contribute to tumour development. Our research group found that LINC00052 expression was repressed during the formation of breast cancer (BC) multicellular spheroids. Intriguingly, LINC00052 precise role in BC remains uncertain. We explored LINC00052 expression in BC patients` RNA samples (TCGA) in silico, as well as in an in-house patient cohort, and inferred its cellular and molecular mechanisms. In vitro studies evaluated LINC00052 relevance in BC cells viability, cell cycle and DNA damage. Results. Bioinformatic RNAseq analysis of BC patients showed that LINC00052 is overexpressed in samples from all BC molecular subtypes. A similar LINC00052 expression pattern was observed in an in-house patient cohort. In addition, higher LINC00052 levels are related to better BC patient´s overall survival. Remarkably, MCF-7 and ZR-75-1 cells treated with estradiol showed increased LINC00052 expression compared to control, while these changes were not observed in MDA-MB-231 cells. In parallel, bioinformatic analyses indicated that LINC00052 influences DNA damage and cell cycle. MCF-7 cells with low LINC00052 levels exhibited increased cellular protection against DNA damage and diminished growth capacity. Furthermore, in cisplatin-resistant MCF-7 cells, LINC00052 expression was downregulated. Conclusion. This work shows that LINC00052 expression is associated with better BC patient survival. Remarkably, LINC00052 expression can be regulated by Estradiol. Additionally, assays suggest that LINC00052 could modulate MCF-7 cells growth and DNA damage repair. Overall, this study highlights the need for further research to unravel LINC00052 molecular mechanisms and potential clinical applications in BC.

## Introduction

Cancer is a group of diseases with a high incidence and mortality rate worldwide. Among several cancer types, Breast cancer (BC) is the primary cause of cancer-related deaths in women globally [1]. Despite the progress made in diagnosing and treating the disease, it is crucial to conduct in-depth research to understand its fundamental aspects. Such studies are a critical and urgent need. BC is a complex and heterogeneous disease resulting from distinct biological and molecular characteristics at both cellular and intra-tumoural levels. According to the PAM50 classification, BC can be categorized into five major subtypes based on molecular profiles: normal-like, luminal A, luminal B, HER2+, and basal-like. Within these molecular subtypes there are well-established biological differences; for instance, it is known that the luminal subtypes have better prognosis and are less aggressive than HER2+ and Basal-like subtypes [2,3].

Scientists have realized that cancer results from a series of genetic and genomic events that occur during the progression from normal tissue to advanced disease. Recently, molecular platforms for gene expression profiling have been extensively employed to find cancer biomarkers and targets for treatment. Identifying long non-coding RNAs (lncRNAs), RNA molecules exceeding a length of 200 nucleotides with low coding potential has become an essential aspect of research. These molecules play a substantial role in regulating diverse cellular mechanisms at both cytoplasmic and nuclear levels, making them a vital factor in understanding various cancer hallmarks [4]. Among several examples, we find HOTAIR (direct metastasis), PANDA (DNA damage repair), MEG3 (proliferation), and NKILA (evasion of the immune system) [5–7].

One of such lncRNA that acts as a regulator of the cancer phenotype is LINC00052 (Long Intergenic Non-Coding RNA 00052, ENCODE nomenclature hg38) [8]. The gene for this RNA is localized between AGBL1 and NTRK3 genes as an anti-sense strand within an intron of the gene lncRNA-NTRK3, with an independent promoter. In addition, LINC00052 has processing and recognition sites for the splicing machinery, which in turn is protected by a 3´- polyA tail [9]. LINC00052 function has been a topic of debate, as it has been found to act as a cancer promoter and a tumour suppressor in different tumour types. This discrepancy could be attributed to the fact that the roles of this RNA are highly dependent on the cellular environment and contextual factors. LINC00052 has been shown to bind or sequester microRNAs and exhibit a functional sponge function, indicating its potential role in regulating gene expression. In this regard, LINC00052 acts as a suppressor gene, at least in hepatocarcinoma [10,11], pancreatic cancer [12], gliomas [13,14], and possibly breast cancer [15–18], while remarkably, it appears to function as an oncogene in gastric cancer [19].

The understanding of the role of LINC00052 in breast cancer is currently limited, and there is some contradictory data. In 2016, Mingming and colleagues reported a decrease in its expression in patients with triple-negative BC [15]. In addition, Mingming’s findings suggest that that vimentin levels regulate LINC00052 in BC. In accordance, Pacheco-Marin and Muñoz-Galindo also reported decreased levels of LINC00052 in three-dimensional breast cancer multicellular spheroids, which mimics the biology of avascular tumours *in vivo*, as the cells become more aggressive [16,17]. Similarly, our research group showed that LINC00052 is associated with improved overall survival in breast cancer patients and that LINC00052 inhibits BC cell migration through modulation of the cytoskeleton and NOTCH/β–catenin/NFkB signalling pathways [20]. On the other hand, Salameh and colleagues reported a positive correlation between LINC00052 expression and HER3/ErbB3 in BC cells. These authors also found that LINC00052 promotes cancer growth by activating the HER3 signalling pathway [21]. Similarly, Dong and collaborators showed that LINC00052 promotes BC cell progression and metastasis by sponging mir-145-5p to modulate TGFBR2 expression [22]. More recently, Huang and colleagues reported that LINC00052 is highly expressed in BC and promotes proliferation, migration, and invasion of these cells through the miR-548p/NOTCH2/Pyk2 axis [23]. Given these findings, it is evident that further research is required to complete the framework and better understand the role of LINC00052 in BC fully.

## Methodology

### In silico approaches

#### Analysis of BC patients’ survival regarding LINC00052 expression

Km-plotter tool was used to analyse the impact of LINC00052 expression (mRNA and RNAseq chip databases) on breast cancer patients overall survival [24]. Analyses were performed using the tool’s default parameters, as well as restricted to hormone receptors.

#### Study of LINC00052 expression patterns and gene enrichment in different BC subtypes

TCGA (The Cancer Genome Atlas) total gene expression data (FPKM, fragments per kb per million) from free access to breast cancer tissue samples from patients were downloaded from (GDC tool) platform. Data were grouped by molecular subtype into Normal-like, Luminal A, Luminal B, HER2+, and Basal-like with information from cBioPortal [2,25]. Quartiles normalized FPKM from each group. Log2FC (Fold Change; FC ± 2, *p-value* < 0.05) was calculated to investigate differentially expressed genes. Additionally, LINC00052 expression was assessed in each BC molecular subtype, as well as the expression of hormone receptors and/or different reference genes using bc-GenExMiner v5 online tool applying exhaustive expression analysis tool from TCGA and METABRIC dataset [26]. Moreover, BC molecular subtype data were used to perform gene enrichment analysis with GSEA tool (Gene set enrichment analysis, Broad Institute online tool) comparing to KEGG database and using Phenotype as permutation criteria and tool’s default parameters.

R tool (v4.1.1) ‘Heatmap’, ‘correlation’ (cor), and ‘ggpairs’ packages were employed to generate heatmaps and perform correlation analyzes for processes enriched with LINC00052 expression. Enrichment results were compared between BC subgroups.

#### Exploration of LINC00052 interactions and promoter analysis

RNAInter v4.0 platform was employed to evaluate potential molecules that associate with LINC00052, using the gene symbol *Homo sapiens* as species and the tool’s default parameters [27]. On the other hand, putative LINC00052 transcription factors were searched with Li C-Lab KnockTF platform [28] using ‘target gene scanning’ as parameters, Gene Symbol as name type, and an FC value of 1.5. A LINC00052 co-expression analysis was performed on patient samples using starBase v2.0 tool, selecting PanCancer RNA–RNA Co-expression parameter [29].

#### Coding potential of LINC00052 measure

The FASTA sequence of LINC00052 was obtained from the LNCpedia 5 database (1966pb), and the coding potential was measured using the CPC2 tool available online [30,31].

### Experimental assays

#### Analysis of LINC00052 expression in BC patient samples

##### Ethical statement.

The project on FFPE breast tissue was previously approved by the Research Ethics Committee of the Zacatecas General Hospital ‘Luz Cosio Gonzalez’, with number 02222/2020. We used 10-year-old FFPE breast tissue samples kept in the archive of the Hospital’s pathology department, for which it was not necessary to obtain the signed consent of the patients. The control samples collected came from patients with lipomas, mastitis, and fibroadenomas. All protocols were designed and performed according to the principles of the Declaration of Helsinki.

##### Processing of FFPE samples and RNA extraction.

Ten 5 µm microtome sections were obtained from each FFPE sample. After deparaffinization, the tissue was processed for RNA extraction following the Trizol method.

Before following the supplier’s protocol for total RNA extraction from tissue, the samples were incubated overnight at 50°C, with shaking at 400 rpm with 250 µL of proteinase K buffer (50 mM Tris pH 8.0, 400 mM NaCl, 2 mM EDTA, 4% SDS) and 50 µL of proteinase K (20 mg/mL) (ThermoFisher Scientific, CA, USA. Cat No. AM2548). At the end of the protocol, the RNA concentration and purity were determined using a NanoDrop One Spectrophotometer.

##### cDNA synthesis.

After RNA extraction, the samples were subjected to digestion to remove contaminated DNA using DNAse I (1 U/µL) RNAse Free (ThermoFisher Scientific, CA, USA Cat No. EN0521) according to the manufacturer’s instructions. The cDNA synthesis was performed with the SuperScript™ IV Reverse Transcriptase Kit (ThermoFisher Scientific, CA, USA Cat No. 18091200) and oligo-random hexamer primers under the following conditions, incubation at 45°C overnight, followed by incubation at 70°C for 10 min. The newly synthesized cDNA was stored at −70°C.

##### qPCR conditions.

The qPCR reaction mix was conformed by 1 µL of cDNA, 10 pmol of each primer, water, and 10 µL of the Maxima™ SYBR® Green/ROX Kit (Thermo Scientific, CA, USA Cat No. K0222) in 20 µL of total volume. The qPCR reaction was carried out in the QuantStudio 1 Real-Time PCR System (ThermoFisher Scientific, CA, USA) under the following conditions: 2 min at 50°C, 2 min at 95°C, followed by 45 cycles at 95°C for 15 s, 57.5°C for 15 s, and 72°C for 1 min. The samples with Ct above 39 were removed. The primers were designed using the sequence reported for each gene in the NCBI database (Supplementary Table S1). The relative expression of the LINC00052 gene was determined using the Livak and Schmittgen method [32], Rn = 2−(ΔΔCq), normalizing with the GAPDH gene.

### Cell culture and LINC00052 knockdown

The MCF-7 breast cancer cell line (‘luminal A’) was cultured in DMEM medium supplemented with 5% Fetal Bovine Serum (FBS) at 37°C in a 5% CO_2_ atmosphere. Two shRNAs targeting the sequence of LINC000052 (shA and shB) were used to inhibit LINC00052 expression in MCF-7 cells (shLuc was employed as control) as previously reported [20].

### Analysis of LINC00052 expression upon estradiol treatment

Three breast cancer cell lines were used: two ER+ (MCF-7 and ZR-75-1) and one ER- (MDA-MB-231). For in vitro assays 1 × 10^5^ MCF-7 cells, 3 × 10^5^ ZR-75-1 cells, and 2 × 10^5^ MDA-MB-231 cells were seeded with the complete medium in 35 cm Petri plates, washed twice 24 hr later with PBS 1X and withdrew of phenol red for 5 days by culturing in phenol red-free medium containing 5% of charcoal-stripped foetal bovine serum. Next, the cells were treated with 10 nM Estradiol (Sigma) for 24 h. Control cells were kept with a complete medium without phenol red and diluent at the same concentration as estradiol-treated cells. Total RNA was extracted from 3 to 6 independent experiments, and LINC00052, pS2 and GAPDH expression were analysed by qPCR employing specific oligonucleotides for each gene (Supp. Table S1) according to Power SYBR® Green PCR Master Mix protocol (Cat. No. 4368577, Thermofisher, CDMX).

### Cristal violet cell viability assay

Twenty thousand MCF-7-shLuc, -shA and -shB cells were seeded by duplicate in 48-well plates. Cells were allowed to grow for 1, 2, 4, 6 and 8 days with 1% and 10% FBS. After each time point, cells were stained with 80 μL of 0.05% crystal violet solution for 20 min at room temperature and washed with tap water. After drying, stained cells were recovered with 400uL of 33% an acetic acid solution, and 100uL of each well were read in 96-well plates on a DTX 880 multi-detector at 570 nm.

### COMET DNA damage analysis

One hundred and fifty thousand MCF-7-shLuc, shA and shB cells were seeded in 12-well plates (by replicate). Next day, 1 mL of DMEM-5% FBS with or without 10 ng/μl Mitomycin C (MMC, Cat. M4287) was added. After 24 h, the cells were scrapped off with PBS 1X, and a 1:10 dilution was made (approximately 15,000 cells). Pelleted cells were soaked in 15 μl complete medium with 60 μl of 1% low melting point agarose (Cat. No. 15517, Gibco BRL). Plugs were mounted on frosted slides previously coated with 100 μl of 1% agarose (Cat. 9539-500 G, SIGMA). Next, another layer of 60 μL of agarose was added to each slide. After polymerization and just before use, slides were placed in lysis buffer (2.05 M NaCl, 100 mM EDTA, 10 mM Trizma Base, pH = 10, 1% TritonX100 and 10% DMSO) for 6 h in cold. Slides were then gently rinsed with electrophoresis buffer (300 mM NaOH and 1 mM EDTA pH = 10) and subsequently evaluated by electrophoresis (20 V/300 mA, 60 min). Later, the slides were carefully washed with 0.5 M TRIS-HCl for five minutes, and finally, 100% cold ethanol was added to allow drying overnight. Slides were covered with 300 μL staining buffer (TE plus 1uL/mL SYBRSafe DNA gel stain (Cat. No. S33102, Invitrogen). Ten fields of each slide were acquired using an epifluorescence microscope 40X objective (AXIO, Zeiss) equipped with a Canon camera. Images were analysed with Komet Analysis system v4.0 software, and the length of migrated DNA (COMET tail), non-migrated DNA (nuclear diameter) and tail moment (COMET tail/nuclear diameter ratio) were evaluated (Supplementary Figure S4) [33].

### Cell cycle distribution analysis

Cell cycle profiles were assessed by flow cytometry, as briefly described here. One million MCF-7-shLuc, -shA and -shB cells were plated in T25 cm tissue culture flasks by replicate. Next day, 4 mL of DMEM-5% FBS with or without 10 ng/μl MMC (Cat. M4287) was added. 24 h later, 4 mL of DMEM-5% FBS medium was added to all cells. Next day, cells were trypsinized and recovered in 1 mL 1X DPBS without Ca2+/Mg2+ by centrifugation. Supernatants were removed and discarded, and pellets were washed twice with 1 mL of 1X PBS. Then, cells were fixed with 500 µl of 4% (v/v) paraformaldehyde for 20 min at room temperature. Next, cells were washed three times with 1X PBS. Sample permeabilization was performed by resuspending pellets in 500 µl of blocking solution [0.05% goat serum (Invitrogen, USA), 0.25% Triton 100X (SIGMA, USA)] diluted in PBS 1X for 30 min at room temperature. Finally, cells were resuspended in 1 mL of DAPI solution (0.25 ug/mL) and analysed with an ATUNE flow cytometer.

### Analysis of LINC00052 expression in cisplatin-resistant MCF-7 cell line

MCF-7 cells were grown on plates 100 mm x 20 mm treated Petri dishes (confluence of 90%) and treated with sublethal doses of cisplatin (Zuridry, Zurich Pharma, QT, MEX, cat. 04–02002 version 03), gradually increasing the concentration of cisplatin treatment, until 1.9 μM was reached and kept during 9 months. Once the cisplatin-resistant subclone was generated, a 1.9 µM cisplatin concentration was used intermittently to preserve drug resistance. Six days before experiment´s performance, cisplatin treatment ceased. The cisplatin-resistant subclone (1.9 µM) was appointed cpR-MCF-7 [34].

The total RNA was obtained from three biological replicates form cpRMCF-7 and wt-MCF-7 cells by the Trizol method (Thermo Fisher Scientific, MA, USA, cat. 15596018). LINC00052 expression was analysed as described previously by qPCR (Suplementary Table S1). Normalized respect to 18S expression level. The experiment was done by triplicate.

### Statistical analysis

Statistical tests were performed with Prism Graph v10.0. Two-way ANOVA (with Bonferroni correction) was used to assess LINC00052 expression changes among the molecular subtypes of BC patients in the TCGA database. Standard error normalized qPCR data and a two-way ANOVA multivariable test was used with a Holm-Sidak correction for the BC in-cohort patient samples. For cell viability assays, data were analysed by applying a two-way ANOVA. A T-student test was employed to evaluate LINC00052 expression upon Estradiol treatment. A group ANOVA and standard error (SE) plotting were used for Comet assays. Finally, examine statistical differences in LINC00052 expression levels between MCF-7 and cpR-MCF-7 cells, qPCR data were normalized for standard error, and a two-way ANOVA test was performed. All experiments were performed at least by triplicate.

## Results

### Higher expression of LINC00052 is correlated to the survival of breast cancer patients

**Figure 1.**
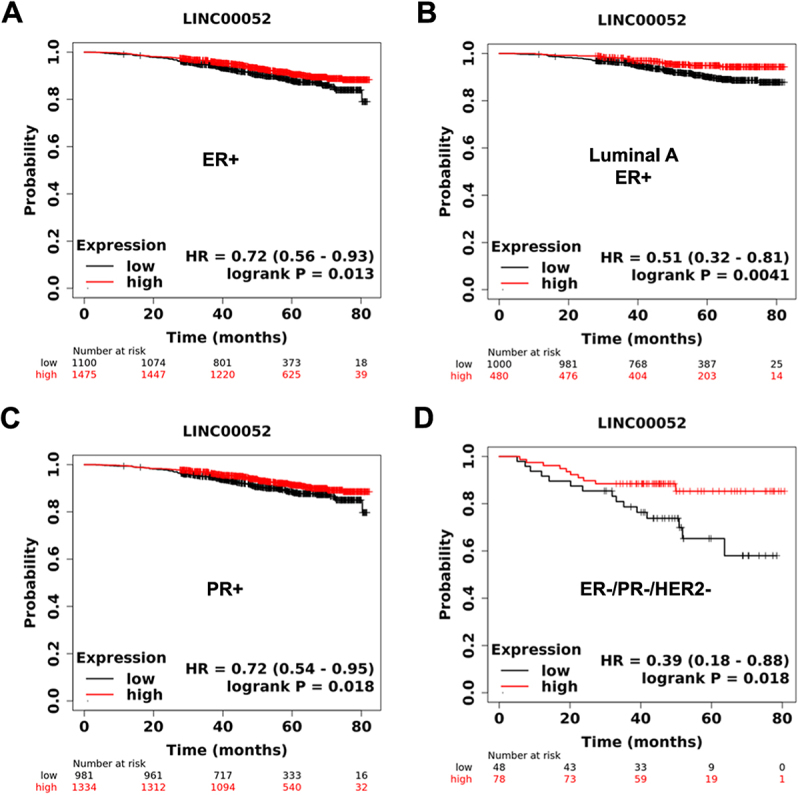
KmPlots survival graphs of breast cancer patients (80 months) with the presence or absence of ER, PR, and HER2 receptors in relation to LINC00052 expression. A) ER+ patient sample group (*n* = 1475); B) Luminal a ER+ patient sample group (*n* = 1480); C) PR+ patient sample group (*n* = 2315); D) TNBC patient sample group (ER-, PR-, HER2-) (*n* = 126). *p* < 0.05.

To determine the clinical relevance of LINC00052, we examined its association with survival in BC patients from RNAseq data using the Km-plotter tool. Oestrogen receptor (ER) and progesterone receptor (PR) positive patients with high LINC00052 expression have more prolonged overall survival than those with low expression (Figure 1A-C). In addition, samples from triple-negative breast cancer (TNBC) patients with higher LINC00052 expression showed increased overall survival rates (Figure 1D). These observations suggest that LINC00052 influences BC patients’ survival and indicates disease aggressiveness, particularly in TNBC samples.

### LINC00052 expression levels across molecular BC subtypes

**Figure 2.**
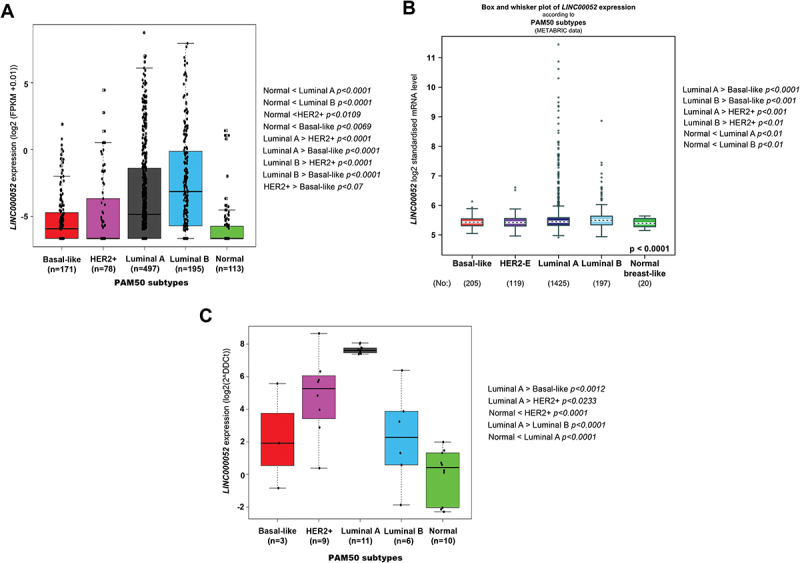
Expression values and changes of LINC00052 in PAM50 breast cancer subtypes. A) Amount of LINC00052 FPKMs expressed in log2, with significant differences shown on the right side for various subtypes of breast cancer (*p* < 0.05) from the TCGA database project. B) Expression of LINC00052 transcript according metabric database. C) LINC00052 expression levels in an in-cohort house of BC Mexican patients samples was assessed by RT-qPCR. Statistical comparisons between the groups and their *p-values* are shown.

To explore LINC00052 differential expression within BC molecular subtypes, a bioinformatic workflow was followed to analyse gene expression data from patient samples in the TCGA international repository (Suplementary Figure S1). The TCGA data FPKMs analysis showed that LINC00052 expression is significantly higher in all molecular subtypes within the BC context, than the Normal-like subtype (Figure 2A). Interestingly, LINC00052 expression levels also vary significantly between different BC subtypes, from which the Basal-like subtype shows the lowest LINC00052 expression level (Suplementary Figure S2a). In addition, LINC00052 expression differences within these subtypes were investigated using the bc-GenExMiner v5 tool with TCGA (Suplementary Figure 2B) and METABRIC data and PAM50 classification (Figure 2B), the results obtained were consistent with what was observed in our analysis, suggesting a higher expression of LINC00052 in the luminal subtypes compared to the HER2+ and basal-like subtypes. Consistent with this, we observed that LINC00052 expression increased in all BC molecular subtypes in an in-house patient cohort compared to healthy tissue (Figure 2C).

**Figure 3.**
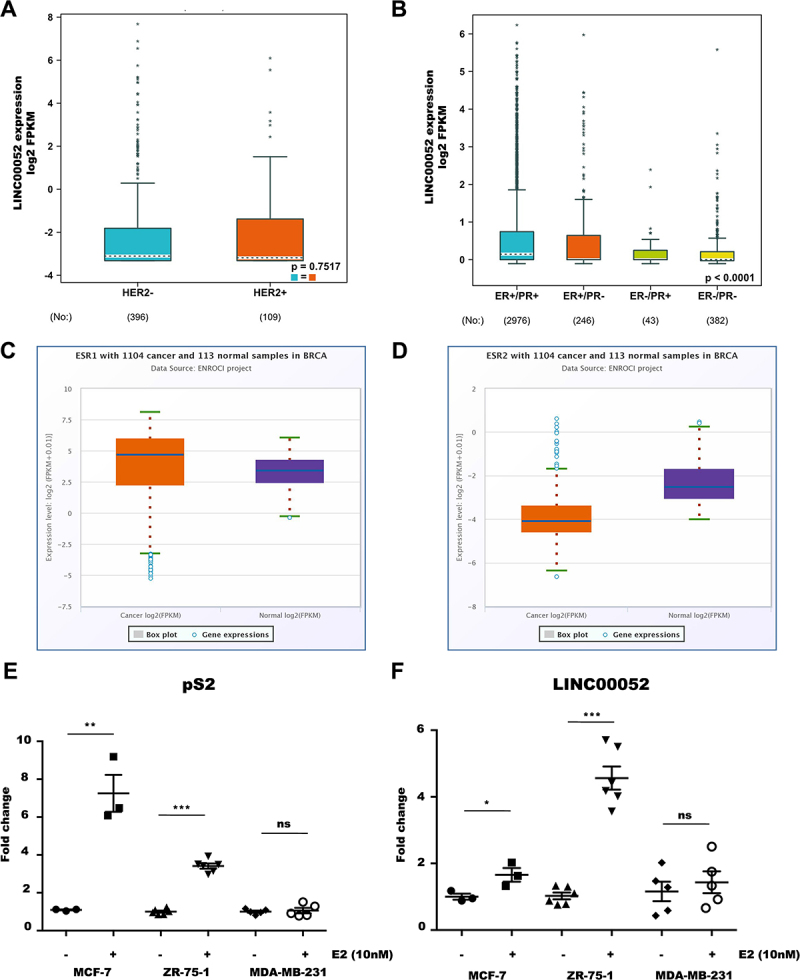
Expression levels of LINC00052 in relation to hormone receptors. A) Expression levels of LINC00052 in log2 FPKMs in HER2+/- samples on a logarithmic scale. B) Expression levels of LINC00052 in log2 FPKMs in ER+/- and PR+/- samples. Statistical significant *p-values* are: ER+/PR+ > ER-/PR- < 0.0001, ER+/PR-> ER-/PR- < 0.01 and ER+/PR+ > ER-/PR+ < 0.01. C) Boxplot of ESR1 expression in log2 FPKMs in 1104 breast cancer samples (cancer) adjacent normal tissue samples (Normal). D) Boxplot of ESR2 expression in log2 FPKMs in 1104 breast cancer samples (Cancer) and 113 adjacent normal tissue samples (Normal). E) Graph of pS2 expression in estradiol-stimulated and unstimulated BC cell lines (10 nM) on the y-axis showing expression levels (Fold change). F) Graph of LINC00052 expression in estradiol-stimulated and unstimulated BC cell lines (10 nM) on the y-axis showing expression levels (Fold change). Results from three to six independent experiments are shown, *p-values* = * 0.01, ** 0.001 and *** 0.0001.

At the same time, we examined at LINC00052 expression levels comparing hormone receptors (ER and PR) and HER2 presence/absence within the TCGA BC tumour samples data using the bc-GenExMiner v5 tool. These results showed that LINC00052 expression does not vary with HER2 expression, but is more highly expressed in the presence of PR receptors (Figure 3A,B).

### Transcriptional regulation of LINC00052 in BC cell lines

Previous analyses suggested a correlation between LINC00052 and hormone receptors expression [16,17]. To explore its transcriptional regulation, we used various bioinformatics tools that would allow us to predict transcription factors that could bind to the LINC00052 promoter site. Predictive analysis done with the Knock TF tool [27] showed that ESR2 (oestrogen receptor β) could be a transcription factor (TF) that positively regulates LINC00052 expression in mammary glands (Supplementary Figure S3a).

In order to support the hypothesis of a relationship between LINC00052 with ESR1 and ESR2 receptors, we further explored data from 1104 patients obtained from TCGA using the StarBase v3.0 algorithm [29]. As shown in Figure 3C,D BC patient samples showed higher levels of ESR1 and lower levels of ESR2 when compared to normal tissue. A positive correlation of LINC00052 with ESR1 expression was observed, in contrast to a negative correlation with ESR2 expression (Supplementary Figure S3B and S3C). In parallel, an analysis done with RNAInter platform showed that one of the transcription factors with which LINC00052 may interact is ESR1 (oestrogen receptor α) as well as AR (Androgen Receptor) (Suplementary Figure S3D). All these results suggest a possible regulation of LINC00052 by ESR1, a hypothesis we decided to confirm.

On the other hand, studies have shown that some lncRNAs have a low coding potential for small peptides, allowing them to have alternative action mechanisms and expression control [35,36]. In this regard, it is unknown whether LINC00052 encodes any peptide. We employed the LINC00052 FASTA sequence to assess this matter bioinformatically and calculated its coding potential. This analysis indicated that the open reading frame (ORF) of LINC00052 can putatively encode a 134 amino acid (aa) peptide; however, at least this predicted potential is very low (coding probability of 0.284677), and it is therefore classified as a long non-coding RNA (Supplementary Figure S3E).

Finally, to know if ESR1 signalling regulates LINC00052, we addressed whether BC cell stimulation with estradiol (10 nM) would modulate LINC00052 expression. Remarkably, after 24 h of estradiol treatment, MCF-7 and ZR-75-1 cells showed an increase in pS2 (estradiol responsive gene) and LINC00052 expression compared to unstimulated cells (control). As expected, when MDA-MB-231 cells were stimulated with estradiol (10 nM), neither ps2 nor LINC00052 expression changed (Figure 3E,F).

### Association between BC enriched cellular processes and LINC00052 expression

**Figure 4.**
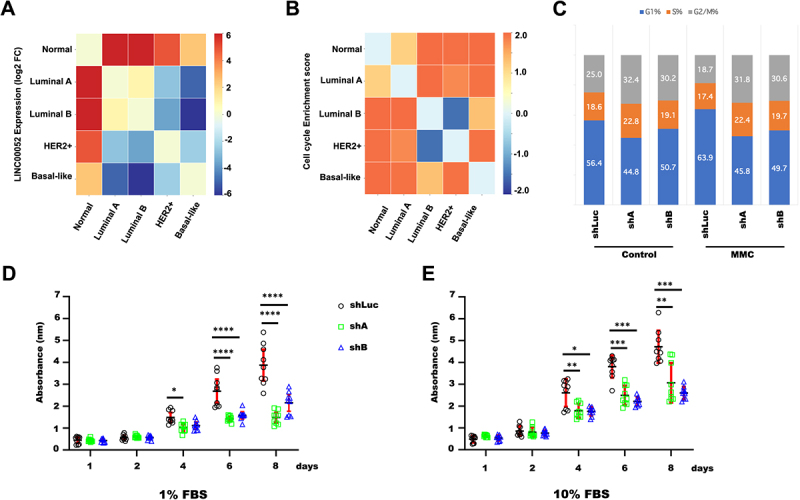
Heatmaps of LINC00052 FC and enrichments of pathways and processes in different molecular subtypes of breast cancer. A) Comparison of log2 FC of LINC00052 among molecular subtypes of breast cancer. B) Score (change value) of cell cycle enrichment. C) Graph shows the average of G1, S, and G2/M cell cycle phases with standard deviation (SD) with and without MMC treatment (24 h) in MCF7 cells, both in control and with LINC00052 levels inhibited with two shRnas. D and E) Graphs show shLuc, shA, and shB cell lines viability (absorbance 580 nm) upon growth in a 1% FBS (D) condition or 10% FBS (E) during indicated time points (1, 2, 4, 6, and 8 days). Results from three to independent experiments are shown, *p-values* = * 0.01, ** 0.001, *** 0.0001 and **** 0.00001.

**Figure 5.**
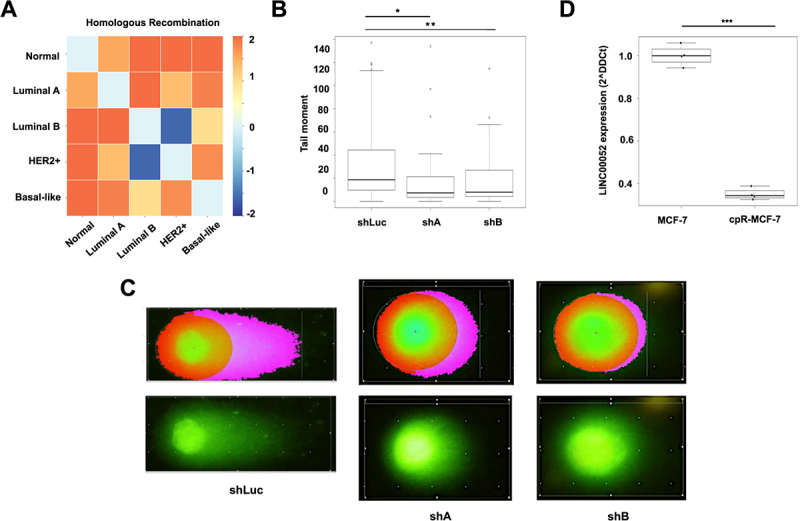
A) Heatmap of homologous recombination process enrichment score. B)Box plot showing the quantification of comet tail moment in COMET assays. C) Pictures represent cells in the comet assays, on the left side observed with komet analysis software, while on the right side, images represent cells directly under an epifluorescence microscope at 40X magnification. Results from three independent experiments are shown: shLuc vs. shA: *p-values* = * 0.0000165 and shLuc vs. shB: ** 0.003. D) Graph shows LINC00052 expression (2^-DDCt) in MCF-7 and cpR-MCF-7 cells, *p-value* = *** 0.0001.

FPKMs obtained from the previously normalized and classified patient samples were used to plot changes in LINC00052 expression (FC) (Figure 4A) and to perform gene enrichment analysis with the Broad Institute GSEA tool [37]. According to the KEGG database, gene expression data from each BC subtype were placed in separate arrays to observe enrichment. These analyses showed that cell cycle (Figure 4B) and DNA repair by homologous recombination (HR) (Figure 5A) were generally enriched in BC and showed specific molecular subtype contrasts. In particular, a comparison between luminal B and HER2+ subtypes with the Basal-like subgroup, where LINC00052 expression is lower (Figure 4A), showed a positive enrichment of these pathways. Moreover, these observations agree with previous findings that suggested that LINC00052 modulates cell cycle and DNA repair mechanism [20].

We analysed cell cycle progression in loss-of-function assays to look at these findings. MCF-7 -shA, -shB and -shLuc cell lines [20] were stimulated with MMC, a cell cycle-dependent double-strand break agent, for 24 h. Data from these experiments showed no significant changes in the percentage of cells in each phase of the cell cycle (Figure 4C).

We then performed a viability assay and analysed MCF-7-shA, MCF-7-shB and MCF-7-shLuc cells growth throughout 1 to 8 days. Interestingly, the results revealed that the proliferation of MCF-7-shA and MCF-7-shB cells was lower when cultured with 1% and 10% SFB, after 4 and 8 days, compared to MCF-7-shLuc cells (Figure 4D,E).

Since homologous recombination was one of the *in silico* enriched processes in BC patients (Figure 5A), we decided to analyse whether MCF-7-shA and B cells exhibited enhanced resistance to DNA damage compared to MCF-7-shLuc cells through a COMET assay (Supplementary Figure S4). Upon stimulation with MMC, it was observed that MCF-7-shLuc cells exhibited a significantly larger tail moment compared to MCF-7-shA and -shB cell lines, suggesting that the inhibition of LINC00052 enhances cellular resistance to MMC-induced genotoxicity Figure 5B,C.

Finally, we looked at LINC00052 expression levels in cisplatin-resistant MCF-7 cells. qPCR results showed that LINC00052 expression decreased in these cells compared to control, suggesting a relation between this lncRNA and resistance to DNA damage (Figure 5D).

## Discussion

Long non-coding RNAs have emerged as essential players in cancer pathology due to their multifaceted functions. Among these, LINC00052 is an intergenic lncRNA that has been shown to be modulated in BC. There are conflicting data on the role of this RNA in cancer, with some studies suggesting that it may function as an oncogene. In contrast, others indicate its potential as a tumour suppressor, therefore we sought to analyse its participation in BC cells [15–18,20–23].

In this study, we conducted *in silico* RNAseq data analysis on BC patients in TCGA and METABRIC. We observed that high LINC00052 expression is associated with increased survival in patients with luminal A, ER+, PR+ and TNBC breast cancer, data consistent with previous reports that showed that high LINC00052 expression is associated with greater overall survival in BC patients [15,20]. Moreover, we observed that LINC00052 expression was similarly modulated in an in-house cohort of BC patients. Overall, ER+ samples displayed elevated levels of LINC00052 expression. Additionally, we identified a significant positive correlation between LINC00052 and ESR1 expression, while a negative correlation was observed between LINC00052 and ESR2 expression. This intriguing result aligns with existing literature highlighting ESR1 expression in tumour cells and ESR2 expression primarily in normal tissue adjacent to the tumour site [38].

Interestingly, when LINC00052 expression is inhibited, ‘Pro-oncogenic action of estradiol_estrogen receptors’ as well as ‘ESR1/ESR2 ratio in breast cancer’ signalling pathways are modulated [20]. Furthermore, we observed that when BC luminal cells were treated with E2, LINC00052 expression increased, which was not the case in basal-like BC cells. Choo and colleagues also reported the regulation of LINC00052 by oestradiol in MCF-7 cells treated (10 nM) for 48 h and analysed by RNAseq [39]. Altogether, these findings suggest there might be a loop between LINC00052 and oestrogen hormone receptors. This type of loop has been described for other lncRNAs, but it is unknown whether a feedback loop exists between LINC00052 and the oestrogen receptor [40]. Furthermore, exploring the impact of other molecules or other microenvironmental conditions on the expression of LINC00052 would be highly worthwhile.

Interestingly, our *in silico* analysis showed that patients belonging to the triple-negative subtype with higher levels of LINC00052 displayed a significant overall survival improvement. Similarly, the ER+ and PR+ subtypes also presented this behaviour, although to a lesser degree. These findings emphasize the need for further investigation into the role of LINC00052 in triple-negative BC patients and its potential as a biomarker.

In parallel, our bioinformatic analyses of patient samples showed that two processes are modified: cell cycle and homologous DNA recombination. Studies have established that a group of lncRNA, especially FOXCUT, MAPT.AS1 and ROCR are modulated across BC molecular subtypes. In particular, a relation between these lncRNA and ER+ and HER2+ hormone receptors has been observed. Furthermore, a link between cell cycle, DNA damage and repair mechanisms, inflammation and apoptosis, and these lncRNA was established differentially among BC molecular subtypes [41].

We previously found that LINC00052 expression probably modulates cell cycle and DNA repair mechanisms [20]. In the present study, we analysed the cell cycle phases in MCF-7 cells with LINC00052 knockdown; however, we did not find significant differences in the distribution of the main cell cycle phases. In contrast, we observed that cells with lower levels of LINC00052 exhibited reduced growth capacity cultures with low and high serum concentrations. On the other hand, *in silico* enrichment analyses suggest an association between homologous recombination and LINC00052 levels, our results *in vitro* demonstrate that reducing LINC00052 levels confers cellular protection against DNA damage. However, whether these cells suffer less DNA damage or repair it more efficiently is still unknown. In particular, when LINC00052 is inhibited, we found that key cell cycle and DNA repair factors, BRCA1 and RAD51 are overexpressed [20]. Consequently, we suggest that when LINC00052 is downregulated, homologous recombination machinery may arrest cell cycle progression until DNA is repaired. BC cells that lack LINC00052 expression may possibly acquire a more aggressive and resistant phenotype to hostile environments. Remarkably, we observed that LINC00052 expression decreased when MCF-7 cells became resistant to DNA-damaging agent cisplatin. In agreement, a previous transcriptome analysis of palbociclib-resistant breast cancer cells showed that LINC00052 expression decreased compared to controls. Together these findings suggest a relationship of LINC00052 with treatment resistance [42].

Recently, Wang and colleagues observed the LINC00052 transcription profile during malignant transformation of normal broncho-epithelial cells (16HBE) by exposure to glycidyl methacrylate (GMA) [43]. Remarkably, these authors observed that LINC00052 is expressed when cell transformation begins and that later transition to a more aggressive state requires its inhibition. These observations highlight the importance of LINC00052 regulation during carcinogenic processes and further support the need to investigate the molecular mechanisms this lncRNA under different physiological and pathophysiological conditions.

## Supplementary Material

Supplemental Material

## Data Availability

Data will be made available on request.
