# Supplementary material for: Decoding LINC00052 role in breast cancer by bioinformatic and experimental analyses
Source: RNA Biol. 2024 Jun 4;21(1):1–11. doi: 10.1080/15476286.2024.2355393 (PMC11152094; doi:10.1080/15476286.2024.2355393)
Supplement: Supplemental Material [file KRNB_A_2355393_SM5355.zip › Supp_Figure_3_copia.pdf]

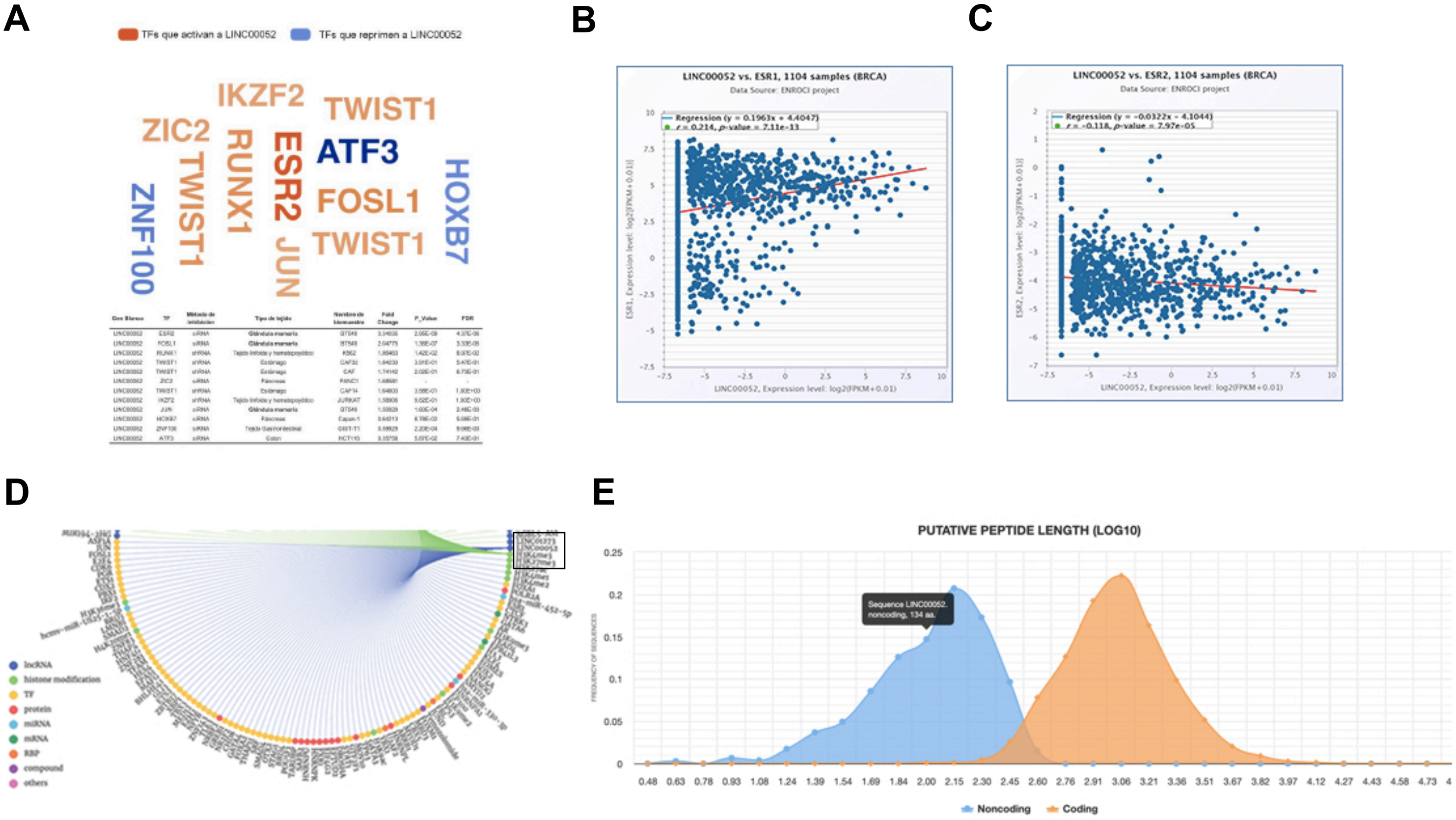
A
TFs que activan a LINC00052
TFs que reprimen a LINC00052
IKZF2
TWIST1
ZIC2
RUNX1
ESR2
ATF3
TWIST1
FOSL1
HOXB7
ZNF100
TWIST1
JUN
B
LINC00052 vs. ESR1, 1104 samples (BRCA)
Data Source: ENROCI project
LINC00052, Expression level: log2(FPKM+0.01)
C
LINC00052 vs. ESR2, 1104 samples (BRCA)
Data Source: ENROCI project
LINC00052, Expression level: log2(FPKM+0.01)
D
lncRNA
histone modification
TF
protein
miRNA
mRNA
RBP
compound
others
E
PUTATIVE PEPTIDE LENGTH (LOG10)
Sequence LINC00052, noncoding, 134 aa.
Noncoding
Coding
