# Supplementary material for: Decoding LINC00052 role in breast cancer by bioinformatic and experimental analyses
Source: RNA Biol. 2024 Jun 4;21(1):1–11. doi: 10.1080/15476286.2024.2355393 (PMC11152094; doi:10.1080/15476286.2024.2355393)
Supplement: Supplemental Material [file KRNB_A_2355393_SM5355.zip › SuppFigure4.pdf]

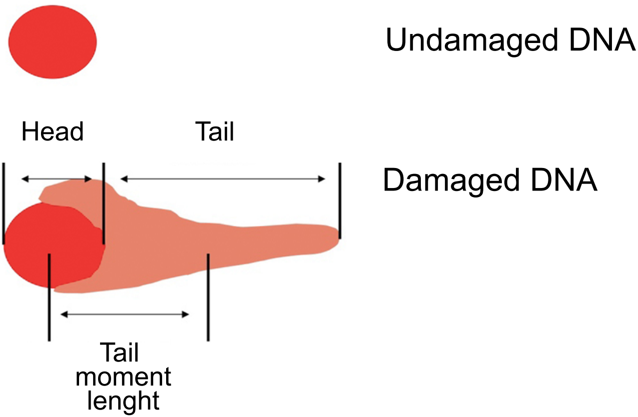

$$\text{DNA damage \%} = \frac{\text{Tail Fluorescence}}{\text{Head Fluorescence}} \times 100$$

$$\text{Tail moment} = (\text{DNA damage \%})\ (\text{Tail moment lenght})$$
