# Supplementary figures and images for: Decoding LINC00052 role in breast cancer by bioinformatic and experimental analyses
Source: RNA Biol. 2024 Jun 4;21(1):1–11. doi: 10.1080/15476286.2024.2355393 (PMC11152094; doi:10.1080/15476286.2024.2355393)

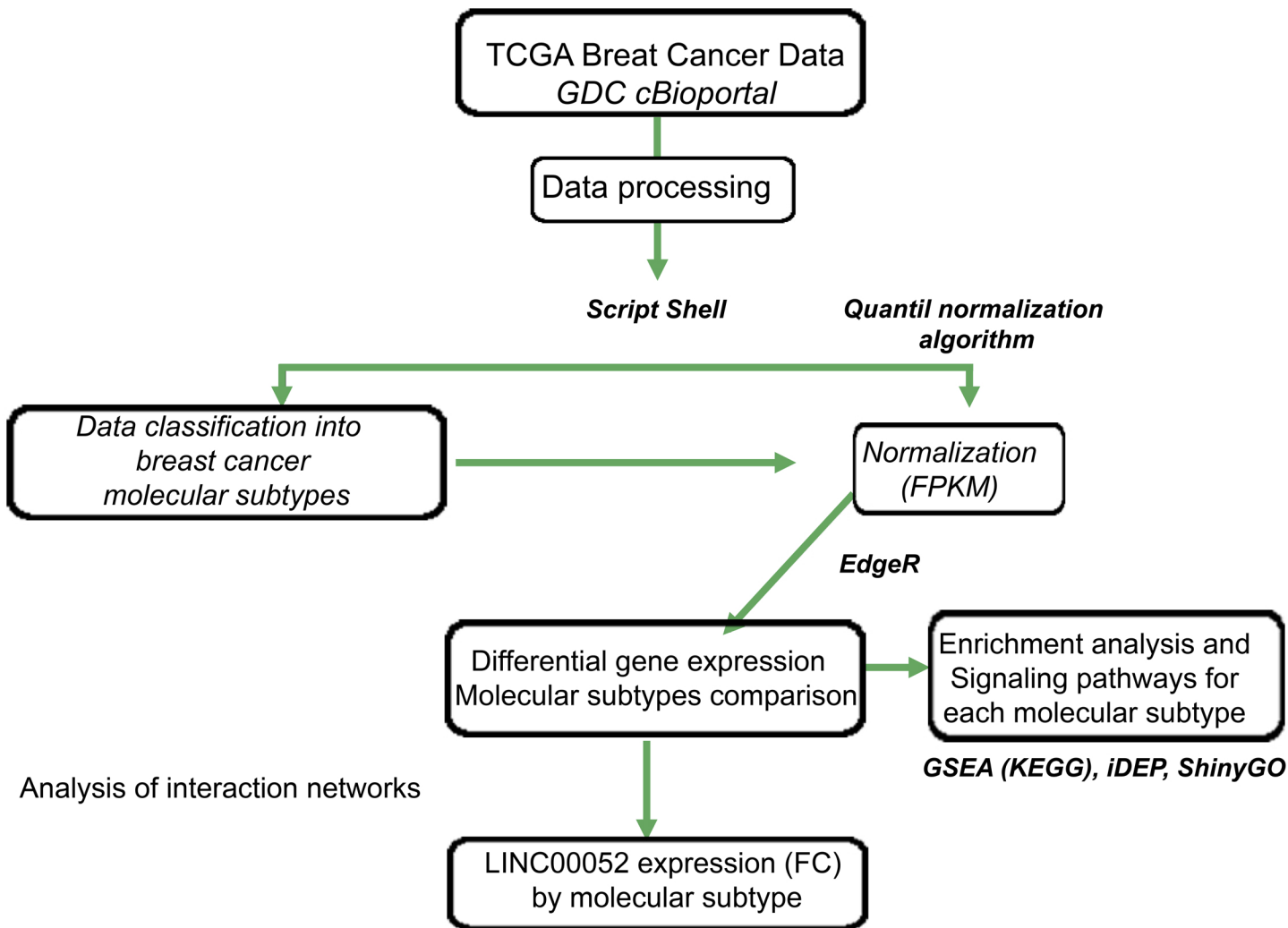

Supplement: Supplemental Material [file KRNB_A_2355393_SM5355.zip › SuppFigure1.pdf]

**A****Log2 FC LINC00052**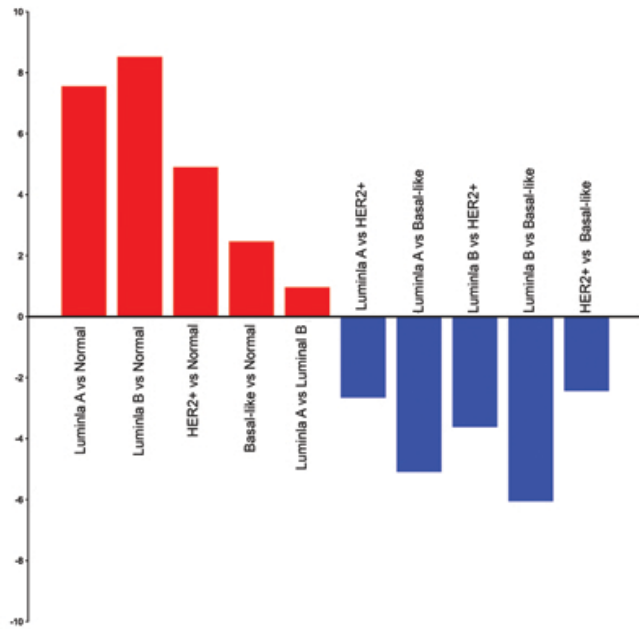**B****LINC000052 expression  
log2 (FPKM +0.01)**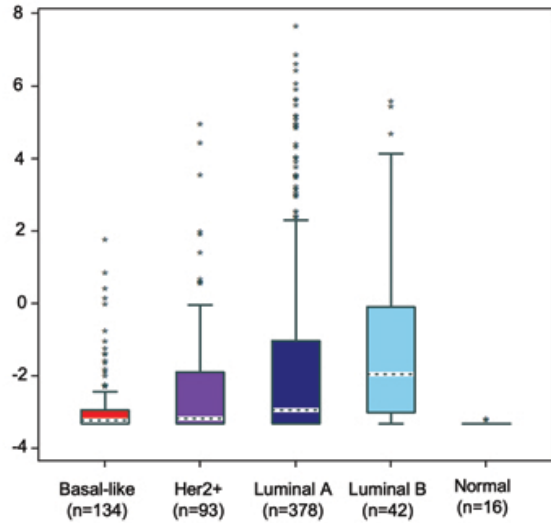

Supplement: Supplemental Material [file KRNB_A_2355393_SM5355.zip › SuppFigure2_copia.pdf]
